# Research on Key Technologies of Dual-Light-Type Photoelectric Colorimetric Method for Phosphate Determination

**DOI:** 10.3390/mi15070821

**Published:** 2024-06-25

**Authors:** Hongzhuang Guo, Hao Zhang, Tingting Sun, Xin Wang, Ping Gong

**Affiliations:** 1 School of Physics, Changchun University of Science and Technology, Changchun 130022, China; guohongzhuang5221@163.com (H.G.); suntingting940113@163.com (T.S.); 2 School of Life Science and Technology, Changchun University of Science and Technology, Changchun 130022, China; zhanghao@cust.edu.cn

**Keywords:** photoelectric colorimetry, phosphate detection, finite element analysis

## Abstract

Phosphate plays a crucial role in microbial proliferation, and the regulation of the phosphate concentration can modulate the fermentation efficiency. In this study, based on Lambert–Beer’s Law and the selective absorption characteristics of substances under light, a dual-light-type photoelectric colorimetric device for phosphate determination was designed. The device’s main components, such as the excitation light path and incubation stations, were modeled and simulated. The primary performance of the instrument was verified, and comparative experiments with a UV-1780 spectrophotometer were conducted to validate its performance. The experimental results demonstrate that this device exhibits a high degree of linearity with an R^2^ value of 0.9956 and a repeatability of ≤1.72%. The average temperature rise rate at the incubation stations was measured at 0.44 °C/s, with a temperature uniformity ≤ ±0.1 °C (temperature set at 37.3 °C). Consistently observed trends in the measurement of 23 CHO cell suspensions using the UV-1780 spectrophotometer further validated the accuracy and reliability of the device’s detection results.

## 1. Introduction

Fermentation is the process wherein microorganisms such as bacteria, fungi, and yeast convert organic substances into useful products under aerobic or anaerobic conditions [1]. Phosphate is an indispensable substance in microbial metabolism, playing a crucial role in microbial proliferation. Firstly, phosphate is a vital component of adenosine triphosphate (ATP). During phosphorylation, microorganisms can convert inorganic phosphate into organic phosphate, generating ATP, which serves as the primary energy source for cellular activities. Secondly, the phosphate concentration is a key factor influencing microbial growth. Both excessively high and low levels of phosphate can negatively impact microbial growth. Thirdly, phosphate is commonly present in buffer solutions used to regulate the pH of microbial growth environments, such as phosphate-buffered saline (PBS). Controlling the phosphate content during fermentation processes can effectively enhance the microbial growth and the product synthesis efficiency.

Common methods for phosphate detection include spectrophotometry, ion chromatography, atomic absorption spectroscopy, microscopy, and spectroscopic analysis. The principles and advantages/disadvantages of each method are summarized in Table 1.

Sean Morgan et al. proposed a microfluidic-based in situ phosphate analyzer, which successfully measured phosphate concentrations in seawater species [6]. While it demonstrated high precision, the instrument’s structure was relatively complex. Heng Xin Zhao et al. suggested a method using fluorescent probes for phosphate detection [7], exhibiting strong specificity, albeit with the complexity of fluorescent reagent synthesis. Hiroshi Aoki et al. developed a phosphate voltametric ion channel sensor based on a gold electrode modified with a self-assembled monolayer of bis-thiourea receptors [8]. Although it avoids sample contamination, the detection results are influenced by the performance of the ion membrane. Tengyue Fang et al. devised an automated analyzer capable of simultaneously detecting silicates and phosphates [9]. Thibaud Rossel et al. proposed a sensor capable of detecting phosphates at neutral pH, albeit with lower accuracy and resolution [10]. Xue Gao et al. introduced a dual-signal biosensor based on a CD-Cu2+ complex [11], exhibiting high accuracy and reliability but requiring sophisticated detection instruments. Peter Franz suggested a biosensor based on thermophoresis for real-time phosphate detection, albeit with complex operation.

As early as 1962, J. Murphy and J. P. Riley proposed a spectrophotometric method for determining phosphate in seawater [12]. Currently, this method serves as the standard approach for phosphate detection in aqueous solutions [13]. Due to slight deviations in the electrical parameters of electronic components after each power cycle, the repeatability of the device is compromised. To enhance repeatability, it requires zeroing and calibration adjustments every time the device is powered on. In response to this issue, this study proposes a dual-light-type photoelectric colorimetric method, which automatically subtracts the background interference, reduces the operational steps, and simplifies the procedure. Comparative experiments with the UV-1780 ultraviolet spectrophotometer validate the stability and reliability of this device.

## 2. Materials and Methods

### 2.1. Reagents and Instruments

The reagents and instruments used were as follows: inorganic phosphorus determination kit (Jilin Base Egg Biotechnology Co., Changchun, China), ultraviolet–visible spectrophotometer (UV-1780, Shimaduz, Kyoto, Japan), quartz ultraviolet cuvette (Starnar 1/Q/10, Shimaduz, Kyoto, Japan), CHO cell suspension (Changchun Biological Products Research Institute Co., Changchun, China).

### 2.2. Overall Design Solution

Colorimetry is a method used to determine the concentration of a substance in a solution by comparing or measuring the color intensity of the solution. As early as the beginning of the Common Era, the ancient Greeks used gallnut solution to determine the iron content in vinegar. In 1795, Russians also used alcoholic solutions of gallnuts to measure the iron content in mineral water. However, colorimetry was formally established as a quantitative analytical method around the 1830s to 1840s. This method leverages the absorption characteristics of colored substances at specific wavelengths for qualitative analysis. The principle is based on the fact that the color intensity of a solution, either inherently or after the addition of a color reagent, is directly proportional to the concentration of the substance. By measuring the intensity of light absorbed by the colored solution, the concentration of the substance in the solution can be determined.

According to the Lambert–Beer law, ammonium molybdate (3.5 g/L) in the reagent reacts with phosphate in the sample under acidic conditions (25 mL/L sulfuric acid) to form phosphomolybdic acid. Phosphomolybdic acid has an absorption peak at a wavelength of 340 nm. To account for background interference, a wavelength of 415 nm is used as the reference. The true absorbance of the sample is determined by the difference between the absorbance values at 340 nm and 415 nm. The absorbance of the test solution should be directly proportional to the concentration of the green complex.

**Figure 1:**
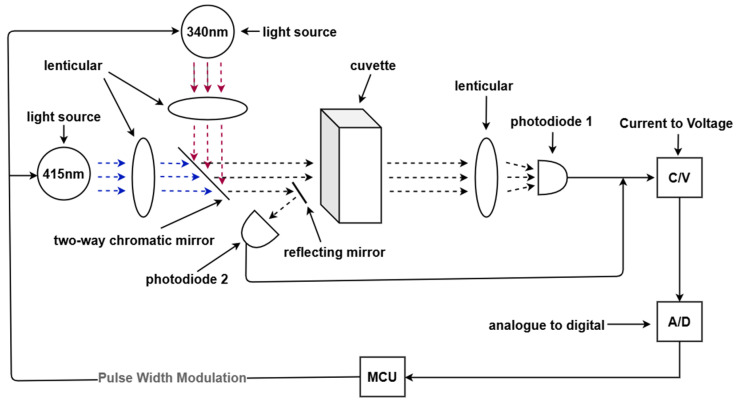
Schematic diagram of detection principle.

This work employs the STM32F103C8T6 microcontroller (STMicroelectronics, Geneva, Switzerland) as the core control chip, the S16838–01MS silicon photodiode (Hamamatsu Photonics, Hamamatsu, Japan) as the photoelectric sensor, and the ADS1256 chip (Texas Instruments, Dallas, TX, USA) as the analog-to-digital converter. As depicted in Figure 1, utilizing a PID algorithm, the microcontroller controls the output light intensity of two sets of PWM to maintain a constant illumination intensity of 340 nm (main wavelength) and 415 nm (auxiliary wavelength) light sources. The light from these two sources converges through convex lenses, then deflects via a dichroic mirror before irradiating a 10 mm optical path quartz cuvette (Starna Cells Inc., Kyoto, Japan). Part of the light is reflected by a mirror, shining onto the photodiode sensor 2, denoted as I_340_ and I_415_, respectively. Another portion of the light passes through the cuvette and converges through convex lenses onto photodiode sensor 1, denoted as E_340_ and E_415_, respectively. By measuring the current change through the silicon photodiode before and after the reaction and calculating the change in light intensity before and after the reaction, the concentration of phosphomolybdic acid in the sample solution can be determined.

Lambert’s law’s [13] mathematical expression is

(1) $$\left\{\begin{array}{c} \mathrm{Ab}=\mathrm{lg}(\frac{1}{T})=K\times b\times c\\ T=\frac{\mathrm{emission}\;\mathrm{light}\;\mathrm{intensity}(E)}{\mathrm{incident}\;\mathrm{light}\;\mathrm{intensity}(I)} \end{array}\right.$$

In the equation, Ab represents the absorbance of the substance, T stands for the transmittance, K denotes the molar absorptivity, which solely depends on the properties of the absorbing substance and the wavelength λ of the incident light, independent of other factors. b represents the thickness of the absorbing layer, with a light path of 10 mm utilized in this design using a standard quartz cuvette. c denotes the concentration of the absorbing substance. E refers to the intensity of the transmitted light, while I represents the intensity of the incident light. As K and b remain constant, let m = K × b, resulting in Formula (2).

(2) $$\mathrm{Ab}=\mathrm{lg}(\frac{I}{E})=m\times c$$

From Equation (2), it is evident that the absorbance of the substance is linearly related to its concentration, while it exhibits a logarithmic relationship with the ratio of incident to transmitted light intensities. Moreover, within a certain range, as the concentration increases, $lg(\frac{I}{E})$ the absorbance linearly increases. Substituting I_340_, I_415_, E_340_, and E_415_ into Equation (2), we can obtain the absorbance at 340 nm wavelength and the absorbance at 415 nm wavelength, respectively, as shown in Equations (3) and (4).

(3) $$Ab_{340}=lg\left(\frac{I_{340}}{E_{340}}\right)$$

(4) $$Ab_{415}=lg\left(\frac{I_{415}}{E_{415}}\right)$$

The difference between the two is the absorbance value of phosphomolybdic acid in the solution. Finally, based on the reaction system between phosphate ions and ammonium molybdate, the concentration of phosphate ions in the original solution can be determined.

**Figure 2:**
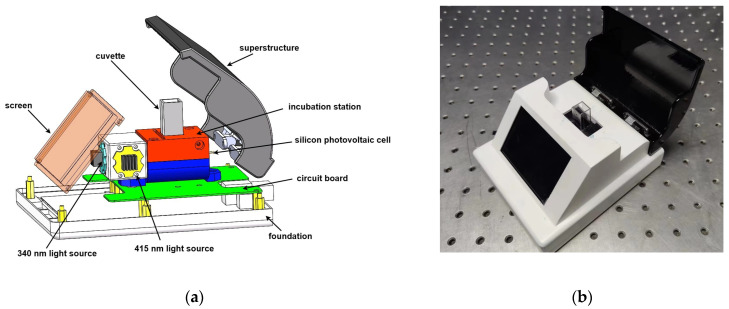
Mechanical design drawings: (**a**) mechanical design drawings; (**b**) actual picture.

The overall mechanical design diagram is shown in Figure 2a, while the physical image is depicted in Figure 2b.

### 2.3. Experimental Methods

#### 2.3.1. Linear Experimental Program

The main components of the inorganic phosphate assay kit are listed in Table 2.

Before starting the experiment, the device was turned on, and the incubation station of the device automatically heated up to 37 °C. The calibration solution in the inorganic phosphate assay kit was diluted to six different concentrations: 1.6 mM, 0.8 mM, 0.4 mM, 0.2 mM, 0.1 mM, and 0.05 mM, using a serial dilution method, with pure water as the 0 mM sample. To minimize sample interference, the experiments were conducted in ascending order of concentration, and the data were recorded accordingly. A graph was plotted with the sample concentration on the *x*-axis and the corresponding absorbance on the *y*-axis, followed by linear fitting. The experimental procedure is outlined in Table 3.

#### 2.3.2. Repeatability Experimental Program

The calibration solutions with concentrations of 0.1 mM and 0.8 mM were subjected to the experimental procedure outlined in Table 3, repeated 10 times for each concentration. The average, standard deviation, and coefficient of variation were calculated for both sets of samples, and the data were recorded accordingly.

#### 2.3.3. Comparison of the Experimental Programs

1. According to the experimental steps outlined in Table 3, the samples of 7 different concentrations mentioned in Section 2.3.1 were measured using both the device described in this study and the UV-1780 UV–Visible spectrophotometer. A graph was plotted with the sample concentration on the *x*-axis and the corresponding absorbance on the *y*-axis, for both instruments, in the same coordinate system. Linear fitting was then performed.
2. Following the experimental steps outlined in Table 3, the CHO cell suspension samples from 23 different batches were measured using both the device described in this study and the UV-1780 UV–Visible spectrophotometer. A graph was plotted with the sample batch number on the *x*-axis and the absorbance of each batch’s sample on the *y*-axis.

## 3. Results and Discussion

### 3.1. Simulation Analysis

#### 3.1.1. Excitation Light Path Simulation

In this study, optical path simulation analysis was conducted using Zemax OpticStudio 19.4 software. The design primarily focused on utilizing dichroic mirrors to reflect or transmit specific wavelengths, enabling the design of two separate excitation paths. These two excitation paths operate independently without interference. The cuvette has a width of 1.0 cm. To avoid the effects caused by light passing through the side walls of the cuvette, the size of the light transmission hole at the incubation station was set to 6 mm × 6 mm. A lens was used to focus the light transmitted through the cuvette onto the detection window of the photodetector to measure the transmitted light intensity. Therefore, the design placed greater emphasis on the intensity of the excitation light, and the uniformity of the excitation light was not a critical requirement.

**Figure 3:**
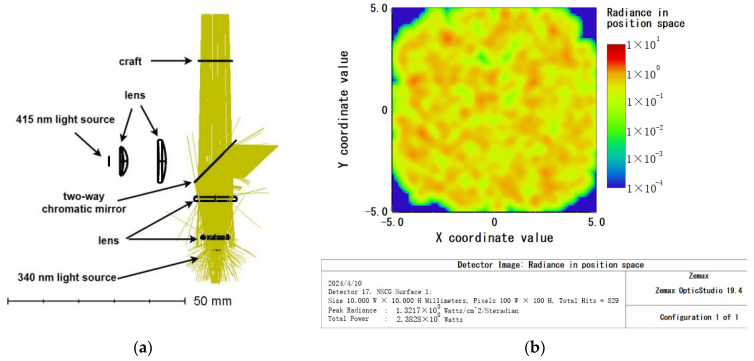
The 340 nm excitation light path: (**a**) optical pathway diagram; (**b**) detector target surface reception diagram.

An IBT-TO46-UV-340 nm LED (manufactured by Shenzhen Ivy Bridge Technology Co., LTD., Shenzhen, China) was utilized as the primary wavelength (340 nm) light source, with a wavelength range from 335 nm to 345 nm. The light passed through two lenses made of H-ZLAF66 material and was then projected onto a rectangular detector through a dichroic mirror. The power of the light source was set at 3 W, and the rectangular detector received a light intensity of 2.38 W, resulting in a calculated light source utilization rate of 79.3%. Refer to Figure 3a,b for details.

**Figure 4:**
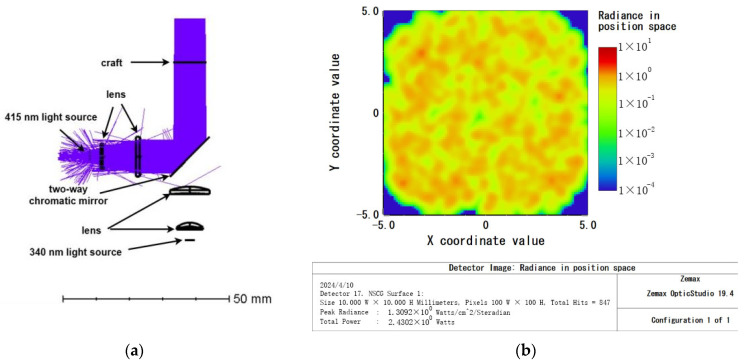
The 415 nm excitation light path: (**a**) optical pathway diagram; (**b**) detector target surface reception diagram.

The C3535U70-UN × 1 LED model (manufactured by SemiLEDs, Taiwan, China) was utilized as the secondary wavelength (415 nm) light source, with a wavelength range from 410 nm to 420 nm. The light passed through two lenses made of H-ZLAF66 material and was then reflected by a dichroic mirror before being projected onto a rectangular detector. The power of the light source was set at 3 W, and the rectangular detector received a light intensity of 2.43 W, resulting in a calculated light source utilization rate of 81.0%. Refer to Figure 4a,b for details.

#### 3.1.2. Temperature Control Module Simulation

**Figure 5:**
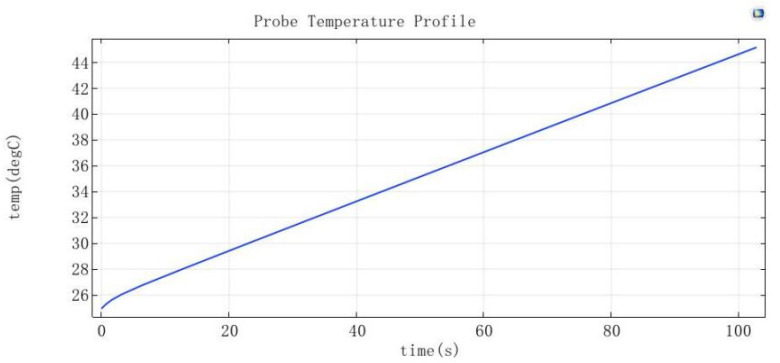
Warming trend profile.

This work used COMSOL Multiphysics 5.4 software to carry out finite element analysis of the temperature performance of the incubation station, due to the better thermal conductivity of 6061 aluminum of about 167 W/(m.K) and the lightweight corrosion-resistant features, it is usually used as the material of the heating device. The device was powered by an external power supply. The rated power of the power supply was 45 W, in order to ensure that the normal operation of the modules under the power supply also retained part of the reserve. The simulation of the incubation station was set to 6061 aluminum, and the initial temperature was set to 25 °C, with the bottom of the 15 W heating tape for heating. In the incubation station and cuvette contact surface at the center of the probe, the probe was set to time T = 0 s when the temperature was 25 °C, and at T = 102.8 s, the temperature was 45.138 °C. Hence, the calculation of the average heating speed of 0.44 °C/s was obtained, as shown in Figure 5.

**Figure 6:**
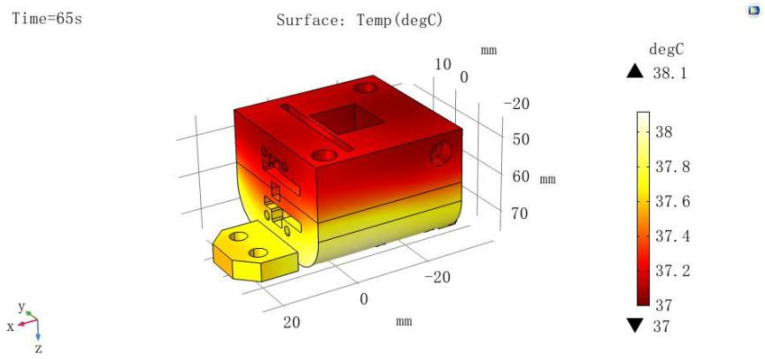
Temperature profile.

According to the simulation results, the incubation station reached the required experimental temperature (37 °C ± 0.5 °C) by T = 65 s. Moreover, the temperature uniformity at the contact point between the incubation station and the cuvette was good, with temperatures ranging from 37.2 °C to 37.4 °C and a uniformity of ±0.1 °C. See Figure 6 for details.

### 3.2. Experimental Results and Discussion

#### 3.2.1. Linear Experimental Results

**Figure 7:**
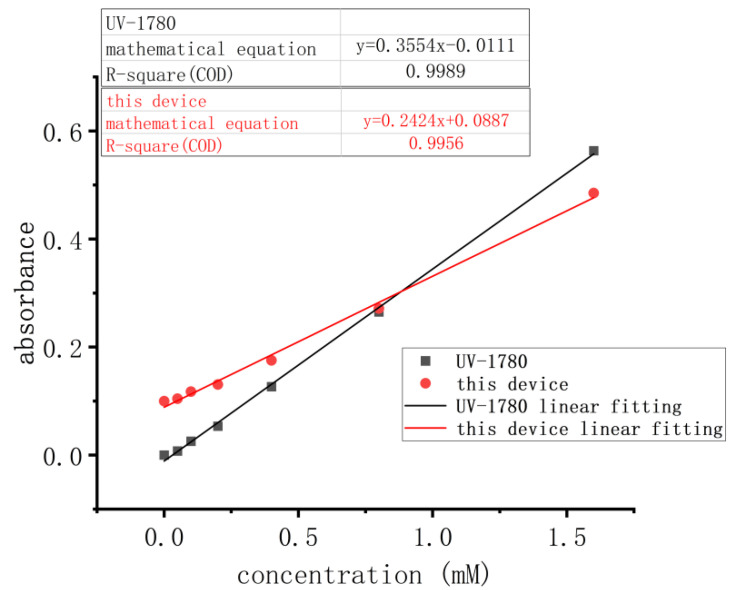
Linear fit diagram.

According to the experimental method described in Section 2.3.1, both the UV-1780 spectrophotometer and the device described in this study were used to test the following seven concentrations. The data obtained are shown in Table 4. Performing linear regression on the two sets of data revealed that the R^2^ value for the UV-1780 spectrophotometer was 0.9989, while for the device it was 0.9956, indicating that the device exhibits good linearity. See Figure 7 for details.

#### 3.2.2. Repeatability Experiment Results

**Figure 8:**
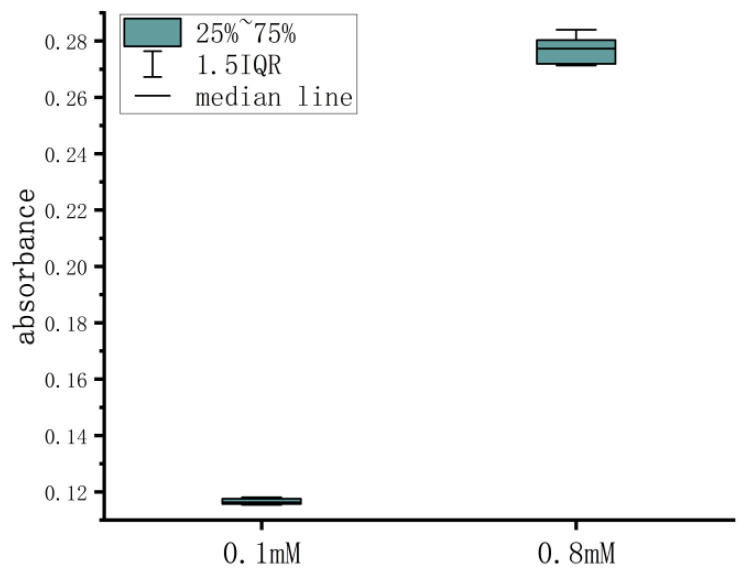
Repeatability test chamber diagram.

The experiment was conducted according to the repeatability experimental plan outlined in Section 2.3.2 for the calibration solutions with concentrations of 0.1 mM and 0.8 mM. The experimental data are recorded in Table 5. The average and standard deviation were calculated for both sets of data. After calculating the coefficient of variation (CV) using CV=SD/mean×100%, the CV values for the two sets of experiments were found to be 0.85% and 1.72%, respectively. To visually observe the results of the repeatability experiments more clearly, a boxplot was created for the two sets of experimental data, as shown in Figure 8. The experimental results indicate that the device exhibits good repeatability, and the repeatability of detecting low-concentration samples is better than that of detecting high-concentration samples.

#### 3.2.3. Comparison of the Experimental Results

**Figure 9:**
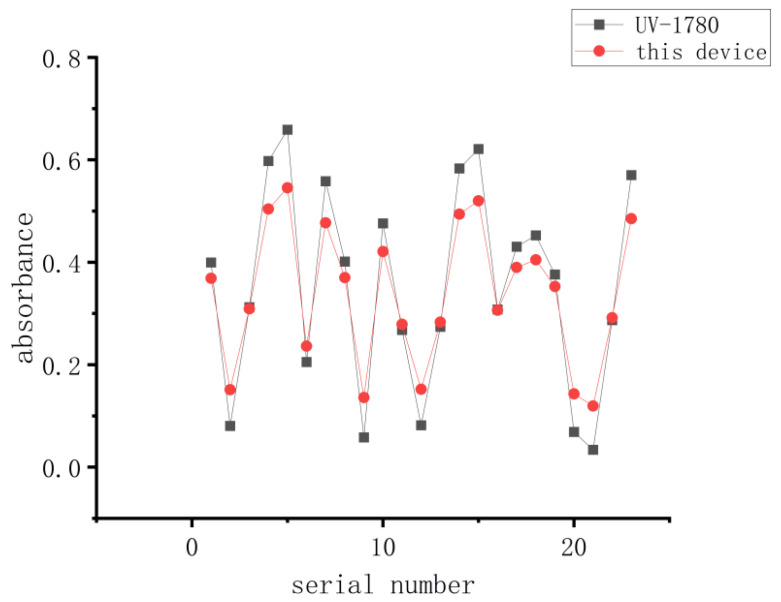
Comparison of experimental diagram.

Using both the UV-1780 spectrophotometer and the device described in this study, the absorbance of 23 CHO cell suspension samples was measured. The test records are shown in Table 6. A graph was plotted with the sample number on the *x*-axis and the absorbance on the *y*-axis, as shown in Figure 9. From the graph, it can be seen that the results obtained by the device and the UV-1780 spectrophotometer exhibited similar trends, indicating that the measurements obtained with the device are stable and reliable.

### 3.3. Discussion

Compared with the UV-1780 spectrophotometer, this device has the advantages of a small size and low price. It can have a built-in calibration curve, with the automatic calculation of the concentration of detected substances. For different kinds of reagents, one can just replace the corresponding band LED excitation light source and recalibrate, achieving a variety of reagents’ detection. The slope of the linear fitting equation of this device is 0.2424, and the slope of the linear fitting equation of the UV-1780 spectrophotometer is 0.3554; hence, the resolution of this device is slightly lower. Improvements can be made by increasing the intensity of the excitation light illumination and increasing the resistance of the resistor in the current–voltage conversion circuit. With the increase in the sensitivity, the repeatability performance of the device may be affected. A comparison with other instruments commonly available on the market is shown in Table 7.

## 4. Conclusions

This paper presents a design scheme for a dual-light photometric colorimetric method for determining phosphate ions, which avoids the conventional spectrophotometer’s “zeroing” and “hundreding” operations, simplifying the procedure and enhancing the overall stability and reliability.

The scientific validity and correctness of the excitation light path design and the incubation workstation design were verified through simulation software. Additionally, a series of experiments were conducted to verify the main performance of the instrument. The device exhibited a linear correlation with R^2^ = 0.9956, repeatability ≤ 1.72%, an average heating rate of 0.44 °C/s for the incubation workstation, and a temperature uniformity of ≤±0.1 °C (at a temperature of 37.3 °C). Through comparative experiments, the results obtained by this device and the UV-1780 spectrophotometer for measuring the absorbance of 23 CHO cell suspensions showed consistent trends, fully demonstrating the accuracy and reliability of the device’s measurements.

## Figures and Tables

**Table 1:** Principles, advantages, and disadvantages of common phosphate detection methods [2,3,4,5].

Detection Methods	Detection Principle	Advantages	Disadvantages	Sensitivity Range
spectrophotometry	Under acidic conditions, phosphate reacts with phenolic reagents such as ammonium molybdate and sodium molybdate to produce colored products, which can be measured for absorbance at specific wavelengths.	Easy and quick to operate.	Vulnerable to interference.	0.01 mg/L~2.0 mg/L
ion chromatography	The phosphate in the solution to be measured is separated from other ions by means of an ion exchange column, and the phosphate concentration is then measured by a detection instrument.	High accuracy and detection of complex samples.	Complex operation and high instrument cost.	0.1 ug/L~10 ug/L
atomic absorption	The phosphate content is determined by measuring the absorption signal of elemental phosphorus in the solution to be tested by atomic absorption spectrometry.	Highly accurate.	Complex operation and high instrument cost.	10 ug/L~50 ug/L
microscopy	Combined with staining techniques under the microscope.	Phosphate morphology and distribution can be observed.	Cumbersome pre-treatment steps and an inability to accurately obtain phosphate concentrations.	Usually used for qualitative and distributional analyses.
spectral analysis	Measurements are made using the absorption or emission properties of phosphates at specific wavelengths, commonly UV–visible spectroscopy, fluorescence spectroscopy, and so on.	High sensitivity and accuracy.	Requires specialized instruments and is costly.	Inductively coupled plasma mass spectrometry can achieve detection limits of sub-micrograms per liter.

**Table 2:** Composition of the anaphylactic phosphorus determination kit.

Kit Composition	Main Components in the Reagent	Concentration
Reagent 1	Oxalate	25 mL/L
Reagent 2	Ammonium molybdate	3.5 g/L
Calibrator	Dipotassium hydrogen phosphate, water-based	1.6 mmol/L

**Table 3:** Experimental step-by-step table.

Serial Number	Experimental Step	Note
1	Add diluted calibrator to cuvette.	3 uL
2	Add reagent 1 to the cuvette.	200 uL
3	Mix well, put into the device, incubate for 3 min, and read absorbance A_0_.	
4	Add reagent 2 to the cuvette.	100 uL
5	After mixing, incubate for 5 min and read the absorbance A_1._	
6	Record ΔA = A_1_ − A_0_.	

**Table 4:** Linear experiment record sheet.

Concentration (mM)	UV-1780 Absorbance	Absorbance of This Device
1.60	0.5635	0.4852
0.80	0.2650	0.2713
0.40	0.1267	0.1753
0.20	0.0536	0.1307
0.10	0.0257	0.1176
0.05	0.0076	0.1044
0.00	0.0000	0.1000

**Table 5:** Record sheet for repeatability experiments.

Serial Number	0.1 mM	0.8 mM
1	0.1176	0.2713
2	0.1154	0.2840
3	0.1181	0.2716
4	0.1177	0.2801
5	0.1165	0.2792
6	0.1156	0.2719
7	0.1157	0.2722
8	0.1160	0.2803
9	0.1173	0.2753
10	0.1161	0.2812
mean	0.1166	0.2767
SD (standard deviation)	0.0010	0.0048
CV (coefficient of variation)	0.85%	1.72%

**Table 6:** Comparative experiment record sheet.

Serial Number	UV-1780	This Device	Serial Number	UV-1780	This Device
1	0.3996	0.3688	13	0.2742	0.2833
2	0.0806	0.1513	14	0.5833	0.4941
3	0.3122	0.3092	15	0.6213	0.5200
4	0.5977	0.5040	16	0.3081	0.3064
5	0.6589	0.5457	17	0.4306	0.3900
6	0.2053	0.2363	18	0.4527	0.4050
7	0.5583	0.4770	19	0.3762	0.3529
8	0.4014	0.3700	20	0.0689	0.1433
9	0.0582	0.1360	21	0.0339	0.1194
10	0.4761	0.4210	22	0.2870	0.2920
11	0.2679	0.2790	23	0.5703	0.4853
12	0.0818	0.1521			

**Table 7:** Comparison table with other instrument parameters.

	This Device	UV-1780	Uicare-300
Sample flux	1	6	32
Sensitivity	0.003 Abs	0.001 Abs	0.01 Abs
Detection range	0.01 mg/L~2.0 mg/L	0.01 mg/L~2.0 mg/L	0.1 mg~2.0 mg/L
Dominance	Low price, small size, easy to operate.	The excitation wavelength is within 340 nm to 900 nm in 1 nm steps.	The sample throughput is relatively large and can be automatically spiked.

## Data Availability

The data that support the findings of this study are available from the corresponding author upon reasonable request.
